# Synthesis and transistor application of the extremely extended phenacene molecule, [9]phenacene

**DOI:** 10.1038/srep21008

**Published:** 2016-02-19

**Authors:** Yuma Shimo, Takahiro Mikami, Shino Hamao, Hidenori Goto, Hideki Okamoto, Ritsuko Eguchi, Shin Gohda, Yasuhiko Hayashi, Yoshihiro Kubozono

**Affiliations:** 1 Department of Electric and Electronic Engineering, Okayama University, Okayama 700-8530, Japan; 2 Research Laboratory for Surface Science, Okayama University, Okayama 700-8530, Japan; 3 Department of Chemistry, Okayama University, Okayama 700-8530, Japan; 4 NARD Co. Ltd. Amagasaki 660-0805, Japan; 5 Research Centre of New Functional Materials for Energy Production, Storage and Transport, Okayama University, Okayama 700-8530, Japan

## Abstract

Many chemists have attempted syntheses of extended π-electron network molecules because of the widespread interest in the chemistry, physics and materials science of such molecules and their potential applications. In particular, extended phenacene molecules, consisting of coplanar fused benzene rings in a repeating W-shaped pattern have attracted much attention because field-effect transistors (FETs) using phenacene molecules show promisingly high performance. Until now, the most extended phenacene molecule available for transistors was [8]phenacene, with eight benzene rings, which showed very high FET performance. Here, we report the synthesis of a more extended phenacene molecule, [9]phenacene, with nine benzene rings. Our synthesis produced enough [9]phenacene to allow the characterization of its crystal and electronic structures, as well as the fabrication of FETs using thin-film and single-crystal [9]phenacene. The latter showed a field-effect mobility as high as 18 cm^2^ V^−1^ s^−1^, which is the highest mobility realized so far in organic single-crystal FETs.

The extension of π-electron networks is one of the most interesting and challenging frontiers of chemistry because π-electrons play important roles in chemical reactions and materials design. Chemistry based on π-electrons has clarified interesting phenomena in diverse fields ranging from life science to materials science. During the past 30 years, the study of organic electronics such as field-effect transistors (FETs) and solar cells based on π-electron network molecules has made rapid progress^1^,^2^,^3^,^4^,^5^,^6^,^7^,^8^,^9^,^10^, and practical organic devices are currently being produced. The successful development of organic electronics is based on progress in π-electron chemistry, and vice versa. Therefore, the chemistry of extended π-electron molecules is currently attracting attention across a wide spectrum of disciplines from pure chemistry to device physics.

Here we briefly survey the present status of organic FETs as an example of recent progress in organic electronics. Field-effect mobilities greater than 10 cm^2^ V^−1^ s^−1^ have been reported in FETs using single crystals of some extended π-electron molecules such as rubrene^11^ and 2,7-dioctyl[1]benzothieno[3,2-*b*][1]benzothiophene (C8-BTBT)^12^. The highest *μ* value realized in an organic single-crystal FET is presently 94 cm^2^ V^−1^ s^−1^ in κ-(BEDT-TTF)_2_Cu[N(CN)_2_]Br (BEDT-TTF: bis(ethylenedithio)tetrathiafulvalene)^13^, using four-terminal measurement. However, the highest *μ* value in single-crystal FETs, recorded using two-terminal measurement, is 18 cm^2^ V^−1^ s^−1^ for rubrene^11^. The smaller *μ* values reported using the two-terminal measurement mode are the result of contact resistance that cannot be eliminated in two-terminal measurement. Recently, organic thin-film FETs using C8-BTBT^14^ have also shown *μ* values as high as 43 cm^2^ V^−1^ s^−1^. This *μ* value is the highest in organic thin-film FETs. All the organic molecules described above are π-electron enriched molecules.

We have attempted to fabricate thin-film and single-crystal FETs using phenacene-type molecules^15^,^16^,^17^,^18^,^19^,^20^,^21^,^22^,^23^. The thin-film FET based on [6]phenacene^18^, with its six fused benzene rings, showed *μ* values as high as 7.4 cm^2^ V^−1^ s^−1^, while a single-crystal FET with [7]phenacene^19^ (7 fused benzene rings), exhibited a *μ* as high as 6.9 cm^2^ V^−1^ s^−1^. Interestingly, the highest *μ* value (20.9 cm^2^ V^−1^ s^−1^) was recorded in a thin-film FET with 3,10-ditetradecylpicene (picene-(C_14_H_29_)_2_)^20^, whose chain of fused benzene rings has two alkyl side chains. Very recently, we synthesized the [8]phenacene molecule, with eight benzene rings, and used it to fabricate thin-film and single-crystal FETs^21^,^22^. The *μ* values were 1.74 and 8.2 cm^2^ V^−1^ s^−1^, respectively, in the thin-film and single-crystal FETs. Since these measurements^15^,^16^,^17^,^18^,^19^,^20^,^21^,^22^,^23^ were performed in two-terminal measurement mode, the realized *μ* values are quite high. We conclude from the above study^22^ that the *μ* value increases with an increasing number of benzene rings, because of the additional π-π interaction.

It is significant to investigate the transistor performance of the FETs using phenacene molecules because phenacene FETs show not only high mobility but also high stability even in atmospheric conditions; the high stability originates from the wide band gap and the deep highest occupied molecular orbital (HOMO) level^23^. Our next synthetic target of a more-extended phenacene molecule than [8]phenacene was naturally [9]phenacene, which should provide a higher *μ* value. However, a sufficient amount of [9]phenacene to fabricate and characterize thin-film and single-crystal FETs had never been synthesized.

In this study we synthesized [9]phenacene, with the success of the synthesis confirmed by time-of-flight mass spectrum, elemental analysis and X-ray diffraction. The crystal and electronic structures were determined with X-ray diffraction analyses of single crystals and powder, and photoelectron yield (PYS) and the absorption spectrum of a thin film. These would be the first experimental evidence of the complete synthesis of the [9]phenacene molecule. Then FET devices were fabricated using thin films and single crystals of [9]phenacene, and their FET properties were fully evaluated. Typical p-channel normally-off FET characteristics were observed in all devices. The best *μ* value in this study was 18 cm^2^ V^−1^ s^−1^ for a [9]phenacene single-crystal FET with a ZrO_2_ gate dielectric, with the measurement performed in two-terminal measurement mode. This *μ* value is comparable to 18 cm^2^ V^−1^ s^−1^ in a rubrene single-crystal FET^11^ that was measured in two-terminal mode, placing the *μ* value in the [9]phenacene single-crystal FET also at the top of the field for two-terminal measurement mode. The threshold voltage, |*V*_th_|, was ~2 V in the device. Thus, a high *μ* value and low-voltage operation were simultaneously realized in the [9]phenacene single-crystal FET with ZrO_2_ gate dielectric. The [9]phenacene thin-film and single-crystal FETs using an electric-double-layer capacitor provided a high *μ* value greater than 1 cm^2^ V^−1^ s^−1^. These results show that the *μ* value continues to increase with an increasing number of benzene rings, implying that increased π-π interaction facilitates channel transport.

## Results

### Preparation of [9]phenacene

**Figure 1:**
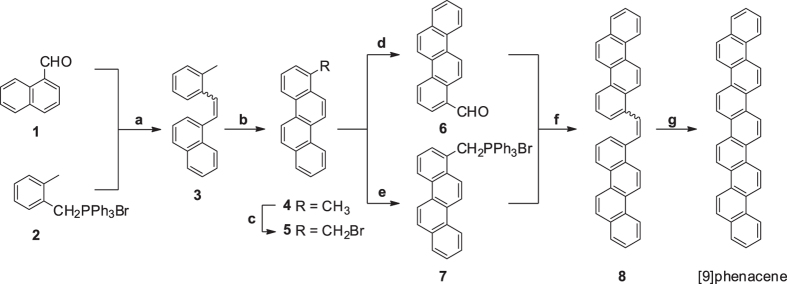
Synthetic route to [9]phenacene.

[9]phenacene was synthesized by the Mallory homologation protocol, which has been used for the synthesis of large phenacene molecules^24^,^25^,^26^, as shown in Fig. 1. Details are described in the Method section and Supplementary Information. This reaction route is composed of a simple repetition of Wittig reactions followed by photocyclization. A Wittig reaction between naphthaldehyde **1** and phosphonium salt **2** afforded naphthyltolylethene **3** as a mixture of *E*- and Z-isomers. The mixture was subjected to photocyclization without separation to produce 1-methylchrysene **4**. The methyl group of compound **4** was brominated with *N*-bromosuccinimide (NBS) in the presence of a catalytic amount of benzoyl peroxide (BPO)^21^. Subsequently, the brominated compound **5** (see Fig. 1) was converted to aldehyde **6** through a Sommelet reaction^27^ and to phosphonium salt **7** by a substitution reaction with triphenylphosphine^21^. The Wittig reaction between compounds **6** and **7** produced dichrysenylethene **8**. The conversion of compound **8** to [9]phenacene was achieved by photocyclization.

**Figure 2:**
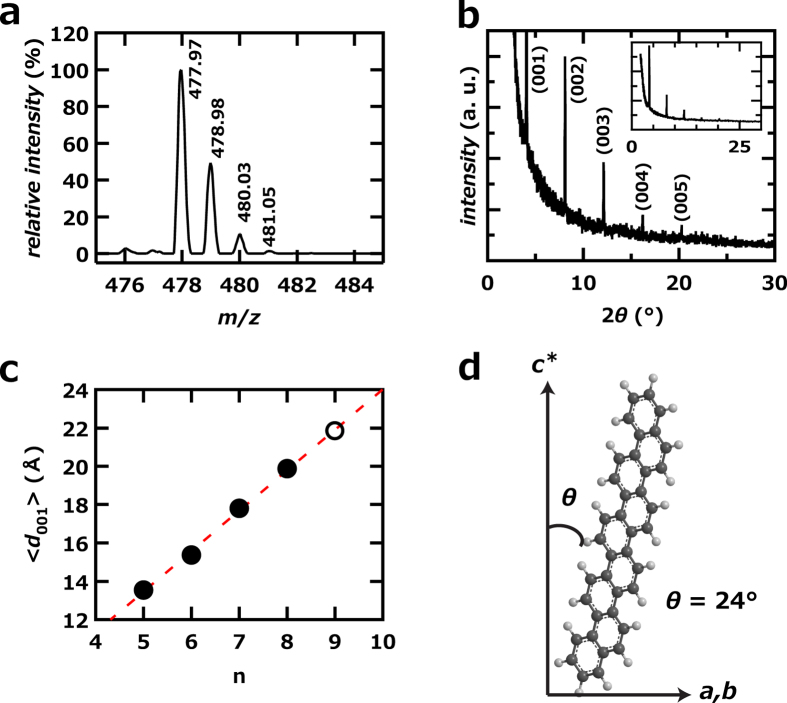
(**a**) Time-of-flight mass spectrum of [9]phenacene sample. (**b**) Out-of-plane XRD pattern of [9]phenacene single crystal. (**c**) <*d*_001_ >- n plot of [n]phenacene. (**d**) Schematic representation showing orientation of [9]phenacene molecule. In (**c**), an open circle refers to [9]phenacene, while solid circles refer to other phenacenes (n = 5 – 8). The <*d*_001_ >’s were evaluated from the out-of-plane XRD patterns of thin films (n = 5 – 8) and crystals (n = 9).

The [9]phenacene molecule was characterized by its MALDI-time-of-flight mass (MALDI-TOF MS) spectrum (Fig. 2a) and the out-of-plane XRD pattern (Fig. 2b) of [9]phenacene single crystals. The MS spectrum showed peaks (m/z = 477.97 for the main peak) ascribable to the [9]phenacene molecule (Fig. 2a); the splitting peaks reflect the natural abundance of isotopes of C and H in [9]phenacene (C_38_H_22_) (details in Methods); table S1 of Supplementary Information lists the observed and calculated *m*/*z*, which shows the good agreement. Figure 2b shows the out-of-plane XRD pattern of a [9]phenacene single crystal. Only (00l) reflections are observed in the X-ray diffraction pattern, which means that the *ab*-plane is parallel to the substrate. The value of the inverse absolute reciprocal of *c*, 1/|c^*^|, which corresponds to the distance between *ab*-layer planes, *d*_001_, was determined from each Bragg peak. When the average value, <1/|c^*^| > = < *d*_001_ > = 21.85(8) Å, for [9]phenacene is plotted as a function of the number, n, of benzene rings, as shown in Fig. 2c, the clear linear relationship confirms that the single crystal is [9]phenacene.

Phenacene molecules with seven to eleven benzene rings have been synthesized by Mallory *et al.*^24^,^25^,^26^. However, these previously synthesized phenacene molecules included substituents to enhance their solubility in organic solvents. These substituents were an essential part of this synthesis of extended phenacene molecules. Therefore, to the best of our knowledge, the [9]phenacene synthesized in this study is the most extended phenacene molecule without substituents. The absence of substituents is important, as their presence on the phenacene core may produce a distorted framework rather than the planar structure^28^ that is required for the formation of the π-electron network that is sought. It is thought that a sufficiently extended π-electron network may make high-channel transport available for high-speed transistors and high-efficiency optical absorption/emission for solar cells and light-emitting diodes. The successful synthesis of un-substituted [9]phenacene in this study should provide π-electron networks with relatively uncompromised planar structures.

### Structure and electronic properties of [9]phenacene

The XRD pattern of a polycrystalline [9]phenacene sample is shown in Figure S1. The XRD pattern was analysed using Le Bail fitting under the space group of *P*2_1_ (monoclinic lattice), and the lattice constants, *a*, *b*, *c* and β, were determined to be 8.844(5), 6.127(3), 22.47(1) Å and 92.72(5)°, respectively. The *c* value is close to 1/|c^*^|, 21.85(8) Å, determined from the out-of-plane XRD pattern (Fig. 2c). The *c*, 22.47(1) Å, increases linearly with increasing n (Figure S2), as shown in the plot of 1/|c*| versus n (Fig. 2c). The lattice constants of phenacene molecules (n = 5 – 9) are listed in Table 1, where the constants of [n]phenacene (n = 5 – 8) are taken from refs 22, 29, and 30. This table shows that the lattice constants for [9]phenacene fit in well.

The inclination angle, *θ*, with respect to c* was evaluated to be 24° by considering the <*d*_001_ > (=21.85(8) Å), determined from the out-of-plane XRD and the van der Waals size of [9]phenacene (long-axis), 23.90 Å. The *θ* value is approximately consistent with the 20°–30° of other [n]phenacene molecules (n = 5 – 8)^22^,^23^. Such a molecular orientation is characteristic of single crystals of phenacene molecules. Further examination of the *θ* value for [n]phenacene is provided in the Discussion section. The orientation of the [9]phenacene molecule with respect to the c* axis is schematically shown in Fig. 2d.

Optical microscope images of thin film and plate-type single crystal of [9]phenacene are shown in Figures S3a and b. Atomic force microscope (AFM) images of a [9]phenacene thin film and a single crystal are shown in Figures S3c and d. The surface of the single crystal is flat and smooth in comparison with that of the thin film; the root mean square (RMS) roughness values in the AFM images (10 *μ*m × 10 *μ*m scale) for thin film and single crystal were 4.4 and 1.7 nm, respectively. A step corresponding to one layer of [9]phenacene is observed on the surface of the single crystal (Figure S3d). The surface features of the [9]phenacene crystal are similar to those of [8]phenacene^22^. We may expect the fabrication of high-performance FET devices with [9]phenacene thin films and single crystals from the similar orientation of the [9]phenacene molecule to other phenacene molecules and the flat surface of the single crystal. Also, the more extended π-framework of [9]phenacene may be superior to [8]phenacene for the channel transport required in an FET device.

**Figure 3:**
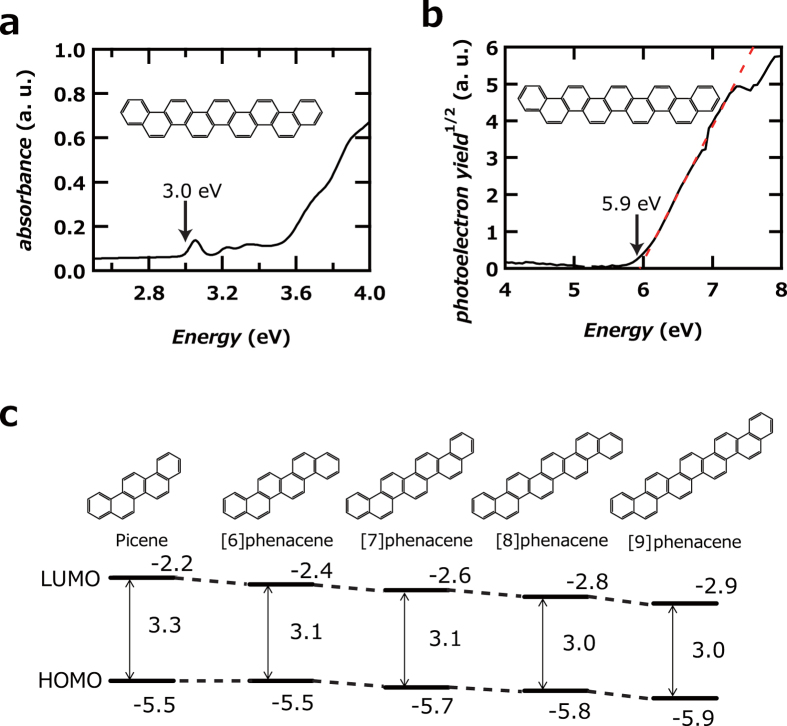
(**a**) Absorption and (**b**) PYS spectra of [9]phenacene thin films. (**c**) Energy diagram of [n]phenacenes. (**d**) Schematic representation of orientation of [9]phenacene molecule in single crystal.

Figure 3a,b show the optical absorption spectrum and photoelectron yield spectrum (PYS) of [9]phenacene thin film. The onset of its optical absorption spectrum was observed at 3.0 eV, while the onset energy of the PYS was 5.9 eV. These imply that the band gap of [9]phenacene thin film is 3.0 eV and the edge of the valence band (or HOMO level) is −5.9 eV; the conduction-band edge (or LUMO level) is −2.9 eV. The edges of the valence band and conduction band of a [9]phenacene thin film are schematically drawn in Fig. 3c, together with the energy diagrams of other phenacene molecules (n = 5 – 8) determined from their optical absorption and PYS spectra. The edge energy of the valence band gradually becomes deeper with increasing n, while the band gap slowly becomes narrower. This slow variation in the energy diagram is characteristic of phenacene molecules, in contrast to the acene molecule^31^. This characteristic can be reasonably explained by the fact that the phenacene molecule does not extend rapidly in the long-axis direction as n increases. The deeper edge of the valence band and the wider band gap than those of acene-type molecules suggest that the phenacene molecule is more stable even under atmospheric conditions, which will be a significant advantage in the deployment of phenacene in practical devices.

### FET characteristics of [9]phenacene thin-film FETs

**Figure 4:**
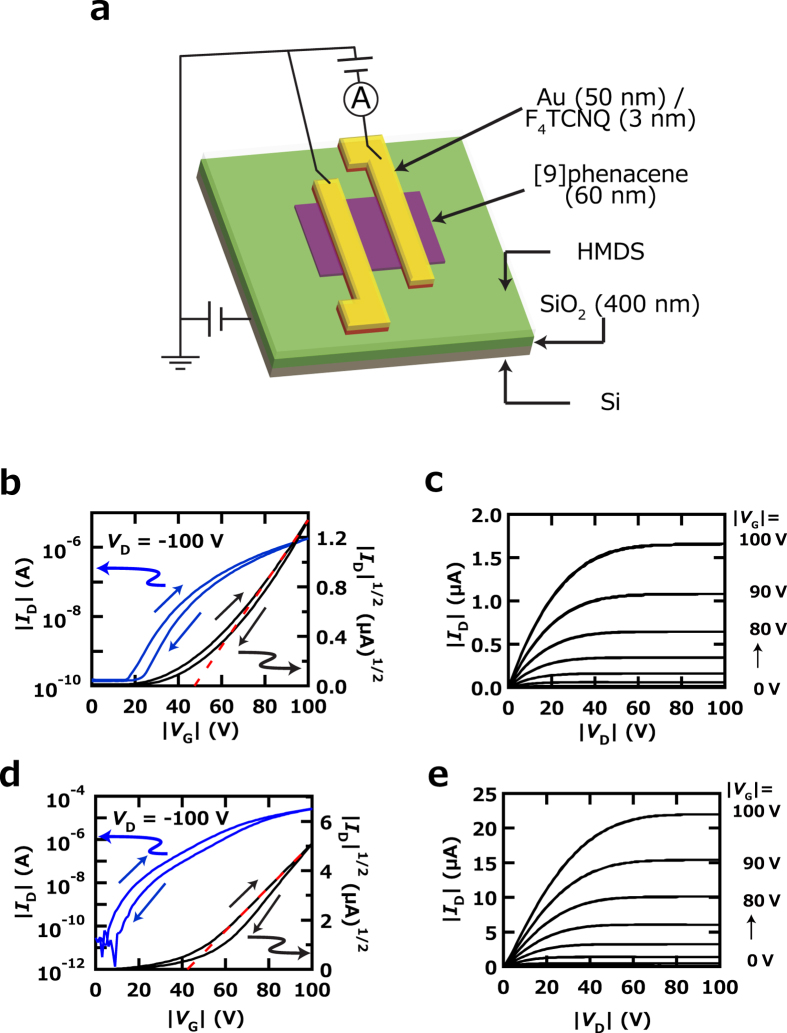
(**a**) Schematic representation of [9]phenacene thin-film FET with an SiO_2_ gate dielectric. (**b**) Transfer and (**c**) output curves of [9]phenacene thin-film FET with an SiO_2_ gate dielectric; amorphous thin film was used for active layer. (**d**) Transfer and (**e**) output curves of [9]phenacene thin-film FET with an SiO_2_ gate dielectric; polycrystalline thin film was used for active layer.

400 nm thick SiO_2_ was used as a gate dielectric in a [9]phenacene thin-film FET device, and the surface of the SiO_2_ gate dielectric was treated with hexamethyldisilazane (HMDS) to produce a hydrophobic surface. The FET characteristics of [9]phenacene thin-film FETs were measured in two-terminal measurement mode. Figure 4a shows a schematic representation of the device structure and measurement mode. Figure 4b,c show the transfer and output curves of the [9]phenacene thin-film FET, exhibiting the typical p-channel normally-off FET characteristics. The FET parameters, *μ*, |*V*_th_|, on-off ratio and sub-threshold swing (*S*) were determined from the forward transfer-curve (Fig. 4b) in the saturation regime (*V*_D_ = −100 V) using the general MOS formula^32^. The *μ*, |*V*_th_|, on-off ratio and *S* were 1.5 × 10^−1 ^cm^2^ V^−1^ s^−1^, 49 V, 1.2 × 10^4^ and 9.7 V decade^−1^, respectively, with the FET properties not strikingly high. The average *μ*, < *μ* > , was 1.2(3) × 10^−1^ cm^2^ V^−1^ s^−1^, based on six devices. The FET parameters for the [9]phenacene thin-film FET with SiO_2_ gate dielectric are listed in Table S2 of Supplementary Information. The <*μ* > value is much smaller than that, 1.2(3) cm^2^ V^−1^ s^−1^, of [8]phenacne thin-film FET with SiO_2_ gate dielectric. The XRD of [9]phenacene thin film showed no Bragg reflections (not shown), implying an amorphous-like thin film.

We tried to fabricate [9]phenacene thin film exhibiting Bragg reflections by changing the condition of thermal deposition of [9]phenacene sample. The Bragg reflections were clearly observed in the XRD pattern (not shown) by cooling the substrate to 278 K during a formation of thin film. The 1/|c*| of thin film was 22.0(5) Å, which is consistent with that, 21.85(8) Å, determined from the single crystal. Figure 4d,e show the transfer and output curves of the [9]phenacene thin-film FET with new thin film exhibiting Bragg reflections, which showed the typical p-channel normally-off FET characteristics, as in Fig. 4b,c. The *μ*, |*V*_th_|, on-off ratio and *S* were 1.7 cm^2^ V^−1^ s^−1^, 42 V, 2.6 × 10^7^ and 3.9 V decade^−1^, respectively, from the forward transfer curve (Fig. 4d). The average *μ*, < *μ* > , was 1.2(3) cm^2^ V^−1^ s^−1^, based on six devices. The FET parameters for the [9]phenacene FETs with new thin films are listed in Table S3 of Supplementary Information. Thus, it has been found that the FET properties are drastically affected by the crystallinity of thin film (or extrinsic factor).

Next we fabricated a [9]phenacene thin-film FET with an electric-double-layer (EDL) capacitor. A schematic representation of the device’s structure and measurement mode is shown in Figure S4a. The gate voltage is applied to the EDL capacitor from the electrode placed on the substrate, *i.e.*, the side gate contact. The transfer and output characteristics of the [9]phenacene thin-film EDL FET are shown in Figures S4b and c, respectively, exhibiting the typical p-channel low-voltage FET operation. The *μ*, |*V*_th_|, on-off ratio and *S* were determined to be 1.1 cm^2^ V^−1^ s^−1^, 2.1 V, 1.8 × 10^3^ and 6.1 × 10^−1^ V decade^−1^, respectively, as averages of the values evaluated from the forward and reverse transfer curves in the saturation regime (*V*_D_ = −1.0 V) using the general MOS formula^32^; the reason that the averaged values are shown as properties of one device is because the hysteresis of the transfer curve (Figure S4b) is too large in the [9]phenacene thin-film EDL FET. This evaluation was also made for the [9]phenacene single-crystal EDL FET in the subsequent section.

The <*μ* > estimated from four [9]phenacene thin-film EDL FETs was 9(2) × 10^−1^ cm^2^ V^−1^ s^−1^, which was lower than that, 8(5) cm^2^ V^−1^ s^−1^, of the [8]phenacene thin-film EDL FET^21^. The FET parameters of the [9]phenacene thin-film EDL FET are listed in Table S4 of Supplementary Information. We fully considered the reason why the properties of [9]phenacene thin-film EDL FET are lower than those of [8]phenacene thin-film EDL FET. The surface-roughness, 4.4 nm, of [9]phenacene thin film is larger than that, 1.7 nm, of [8]phenacene thin-film, which may lead to the lower *μ* in [9]phenacene thin-film EDL FET because the surface region corresponds to the channel formed by EDL capacitor. Namely, the scattering of hole carrier may be larger in [9]phenacene thin film than [8]phenacene.

From the FET properties realized in [9]phenacene thin-film FETs, the quality of thin film has been found to be important for the fabrication of high-performance [9]phenacene thin-film FET. For a realization of high-performance phenacene thin-film FETs, the high crystallinity and flat surface in thin films must be pursued by fully investigating the condition for thermal deposition of phenacene molecules.

### FET characteristics of [9]phenacene single-crystal FETs

**Figure 5:**
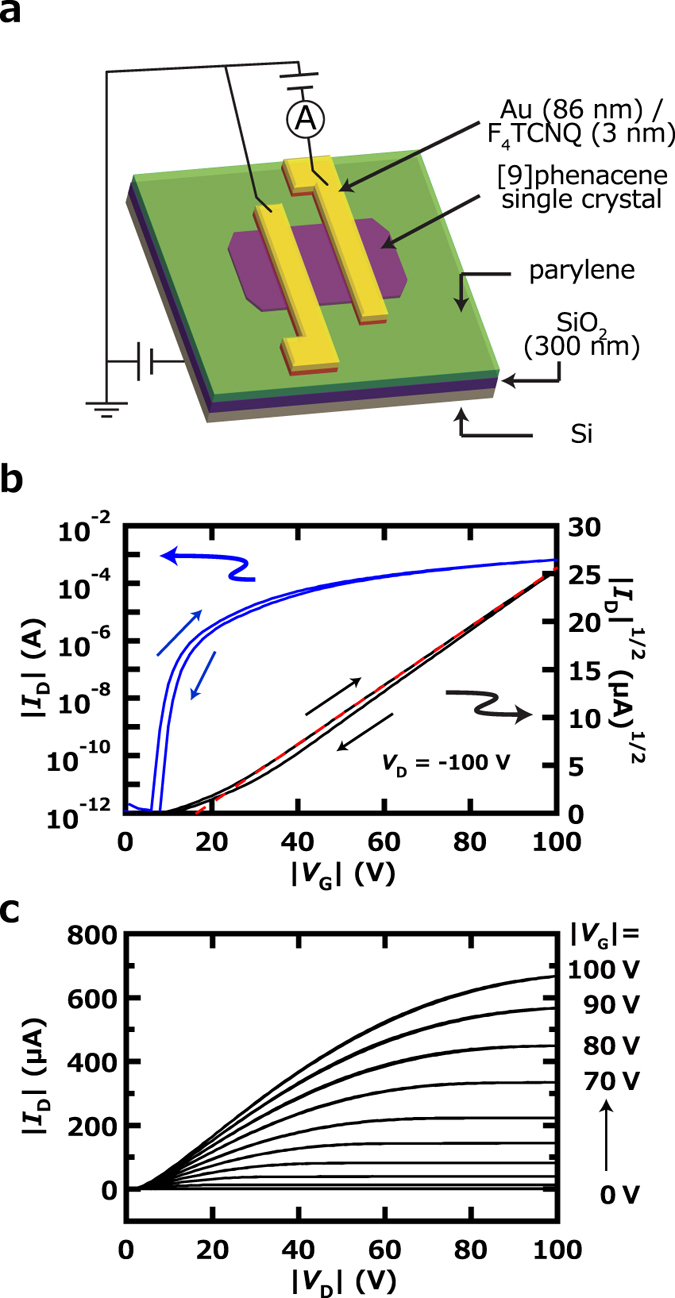
(**a**) Schematic representation of [9]phenacene single-crystal FET with an SiO_2_ gate dielectric. (**b**) Transfer and (**c**) output curves of [9]phenacene single-crystal FET with an SiO_2_ gate dielectric.

300 nm thick SiO_2_ covered with 30 nm thick parylene was used in the [9]phenacene single-crystal FETs. It is well known that parylene provides an excellent hydrophobic surface. The FET characteristics of [9]phenacene single-crystal FETs were measured in two-terminal measurement mode. Figure 5a shows a schematic representation of the device structure and measurement mode. Figure 5b,c show the transfer and output curves of the [9]phenacene single-crystal FET, exhibiting the typical p-channel normally-off FET characteristics. The *μ*, |*V*_th_|, on-off ratio and *S* were determined from the forward transfer-curve (Fig. 5b) in the saturation regime (*V*_D_ = −100 V) using the general MOS formula^32^. The *μ*, |*V*_th_|, on-off ratio and *S* were 10.5 cm^2^ V^−1^ s^−1^, 16.5 V, 5.3 × 10^8^ and 9.0 × 10^−1^ V decade^−1^, respectively, from the forward transfer curve (Fig. 5b). The very small hysteresis is observed in the transfer curves shown in Fig. 5b. The *μ* determined from the reverse transfer curve is 11.6 cm^2^ V^−1^ s^−1^, in consistent with that of that, 10.5 cm^2^ V^−1^ s^−1^, from the forward transfer curve. The hysteresis generally originates from H_2_O in the channel region, but the parylene-coating of the SiO_2_ surface produced a strong hydrophobic circumstance to supress the hysteresis. The *μ* value is very high in comparison with those of other phenacene single-crystal FETs; as described later, a higher *μ* value is realized in a [9]phenacene single-crystal FET with a high-*k* gate dielectric. The <*μ* > of the [9]phenacene single-crystal FET with an SiO_2_ gate dielectric was 8(1) cm^2^ V^−1^ s^−1^ (average of ten devices; see Table 2). The FET parameters obtained from the forward transfer curves for the [9]phenacene single-crystal FET with an SiO_2_ gate dielectric are listed in Table 2, while those from the reverse transfer curves are listed in Table S5.

**Figure 6:**
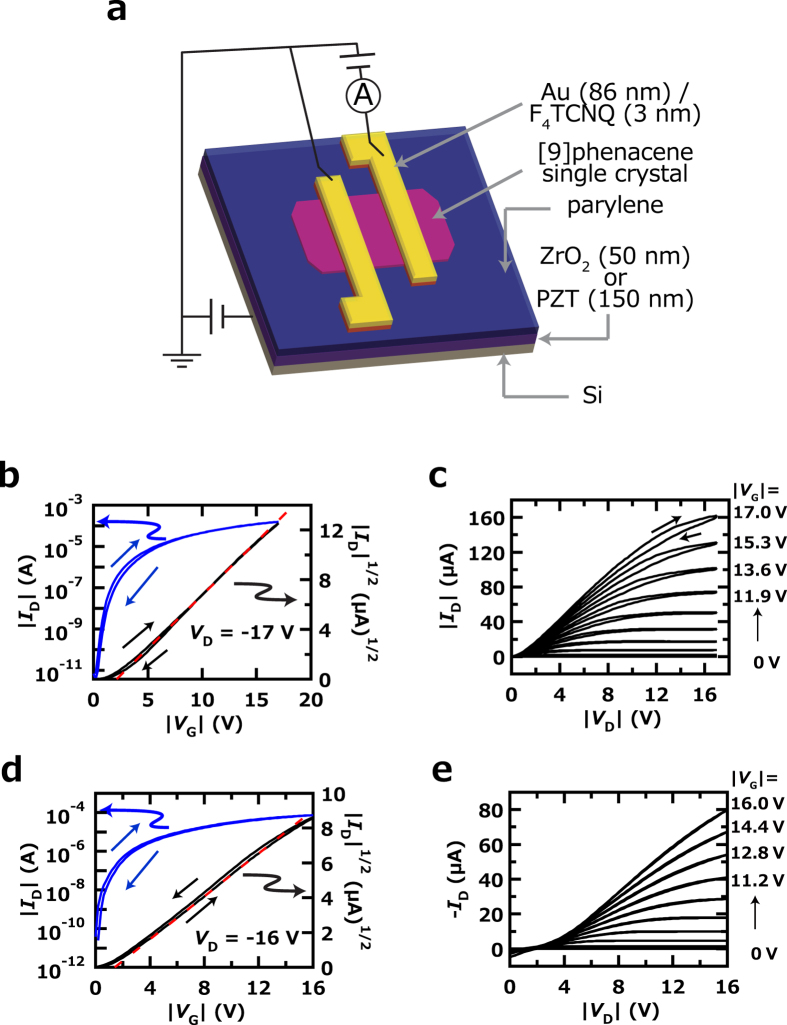
(**a**) Schematic representation of [9]phenacene single-crystal FET with a high-*k* gate dielectric. (**b**) Transfer and (**c**) output curves of [9]phenacene single-crystal FET with a ZrO_2_ gate dielectric. (**d**) Transfer and (**e**) output curves of [9]phenacene single-crystal FET with a PZT gate dielectric.

To realize low-voltage operation in [9]phenacene single-crystal FETs, 50 nm thick ZrO_2_ and 150 nm thick PbZr_0.52_Ti_0.48_O_3_ (PZT) gate dielectrics covered with 30 nm thick parylene were used. The FET characteristics were measured in two-terminal measurement mode. Figure 6a shows a schematic representation of the [9]phenacene single-crystal FETs with ZrO_2_ and PZT gate dielectrics (high-*k* gate dielectrics) to make clear the device structures and measurement mode. Figure 6b,c show the transfer and output curves of a [9]phenacene single-crystal FET with the ZrO_2_ gate dielectric, which exhibit typical p-channel normally-off FET characteristics. The *μ*, |*V*_th_|, on-off ratio and *S* were determined from the forward transfer-curve (Fig. 6b) in the saturation regime (*V*_D_ = −17 V) using the general MOS formula^32^. The *μ*, |*V*_th_|, on-off ratio and *S* were 18 cm^2^ V^−1^ s^−1^, 2.1 V, 2.9 × 10^7^ and 2.1 × 10^−1^ V decade^−1^, respectively. This *μ* value is the highest reported so far in single-crystal FETs with phenacenes (n = 5 – 9). The <*μ* > of the [9]phenacene single-crystal FET with ZrO_2_ gate dielectric was 10(5) cm^2^ V^−1^ s^−1^, as evaluated from five FET devices (Table 3). The average |*V*_th_|, <|*V*_th_| > , was 1.8(6) V, implying low-voltage operation. The lower *μ* values than 10 cm^2^ V^−1^ s^−1^ obtained for two FETs (#3 and #4 in Table 3) originates from the short channel length (50 *μ*m for #3 and 100 *μ*m for #4). If we consider only three devices with long channel length (#1, #2 and #5), the <*μ* > becomes 13(4) cm^2^ V^−1^ s^−1^. These results clearly show that the [9]phenacene single-crystal FET (ZrO_2_ gate dielectric) with long channel length provides a very high FET performance (or high mobility). No or quite small hysteresis is observed in the transfer curves shown in Figure 6b,d. As described previously, the parylene-coating of the high-*k* dielectrics’ surface produced a strong hydrophobic circumstance to disappear the hysteresis.

As seen from Fig. 6c, a slight concavity is observed in the output curves of [9]phenacene single-crystal FETs with ZrO_2_ gate dielectrics, suggesting the possibility of [9]phenacene single-crystal FETs exhibiting higher *μ* values through the lowering of contact resistance. The FET parameters of [9]phenacene single-crystal FETs with ZrO_2_ gate dielectrics are listed in Table 3.

Figure 6d,e show the transfer and output curves of [9]phenacene single-crystal FET with PZT gate dielectric, which exhibit typical p-channel normally-off FET characteristics. The *μ*, |*V*_th_|, on-off ratio and *S* were determined from the forward transfer curve (Fig. 6d) in the saturation regime (*V*_D_ = −16 V) using the general MOS formula^32^. The *μ*, |*V*_th_|, on-off ratio and *S* were 6.2 cm^2^ V^−1^ s^−1^, 1.4 V, 1.7 × 10^7^ and 1.9 × 10^−1^ V decade^−1^, respectively. The <*μ* > of a [9]phenacene single-crystal FET with PZT gate dielectric was 5.5(8) cm^2^ V^−1^ s^−1^, as evaluated from three FET devices. The <|*V*_th_| > was 1.3(3) V, implying low-voltage operation. As seen in Fig. 6e, a large concavity is observed in the output curves of [9]phenacene single-crystal FETs with PZT gate dielectrics. The FET parameters for [9]phenacene single-crystal FETs with PZT gate dielectrics are listed in Table S6 of Supplementary Information. Thus, [9]phenacene single-crystal FETs showed excellent FET characteristics, suggesting the usefulness of their extended π-framework in FET operation. Therefore, the relatively low FET characteristics (*μ* ~ 1 cm^2^ V^−1^ s^−1^) in [9]phenacene thin-film FETs apparently originate in the low quality of thin films, *i.e.*, an extrinsic factor. This was clearly verified from the difference in *μ* value between amorphous and polycrystalline thin films, as described in the previous section.

The transfer and output curves of the [9]phenacene single-crystal FET with an EDL capacitor were measured in two-terminal measurement mode. A schematic representation of the measurement in the [9]phenacene single-crystal EDL FET is shown in Figure S5a. The gate voltage was applied through the Au plate placed on the EDL capacitor (top-gate device structure), unlike that in the [9]phenacene thin-film EDL FET. Figures S5b and c show the transfer and output curves of the [9]phenacene single-crystal EDL FET, exhibiting p-channel FET properties. The *μ*, |*V*_th_|, on-off ratio and *S* were determined to be 1.5 cm^2^ V^−1^ s^−1^, 1.1 V, 4.1 × 10^5^ and 1.3 × 10^−1^ V decade^−1^, respectively, which are the averages of values evaluated from the forward and reverse curves. The <*μ* > was evaluated as 1.2(7) cm^2^ V^−1^ s^−1^ from three EDL FETs. All FET parameters are listed in Table S7; FET number 2 provided Figures S5b and c. Low-voltage operation was realized in the [9]phenacene single-crystal FET with EDL capacitor, but the *μ* was still ~1 cm^2^ V^−1^ s^−1^.

The <*μ* > , 1.2(7) cm^2^ V^−1^ s^−1^, of [9]phenacene single-crystal EDL FET is a little higher than that, 4(2) × 10^−1^ cm^2^ V^−1^ s^−1^, of [8]phenacene single-crystal EDL FET. The reason is still unclear, because the surface-roughness, 1.7 nm, of [9]phenacene single crystal is larger than that, 0.5 nm, of [8]phenacene single crystal. Therefore, the reason may not be so simple as in a thin-film EDL FET.

## Discussion

In this study, the extended benzene-network molecule [9]phenacene was successfully synthesized. This molecule consists of nine fused benzene rings in a repeating W-shaped structure. Few attempts at the synthesis of such an extended phenacene molecule without alkyl side chains have been made because of the difficulties encountered, largely due to the low solubility of the extended phenacene molecule. To our knowledge, no synthesis of [9]phenacene has ever been published. While a patent on the formation of [9]phenacene has appeared^33^, the experimental details are unclear. We used the ‘Mallory homologation protocol’, which consists of the simple repetition of a Wittig reaction followed by photocyclization for the synthesis of [9]phenacene, which is different from the synthetic route in the patent. Our protocol for the synthesis has the advantage of requiring only the simple repetition of an established reaction sequence such as the Wittig reaction and Mallory-photocyclization. Therefore, it can easily be utilized for the synthesis of more extended phenacenes and may become a standard procedure for synthesizing non-substituted [n]phenacenes. In principle, more extended phenacene molecules (n > 9) should also be effectively synthesized using our synthetic protocol.

**Figure 7:**
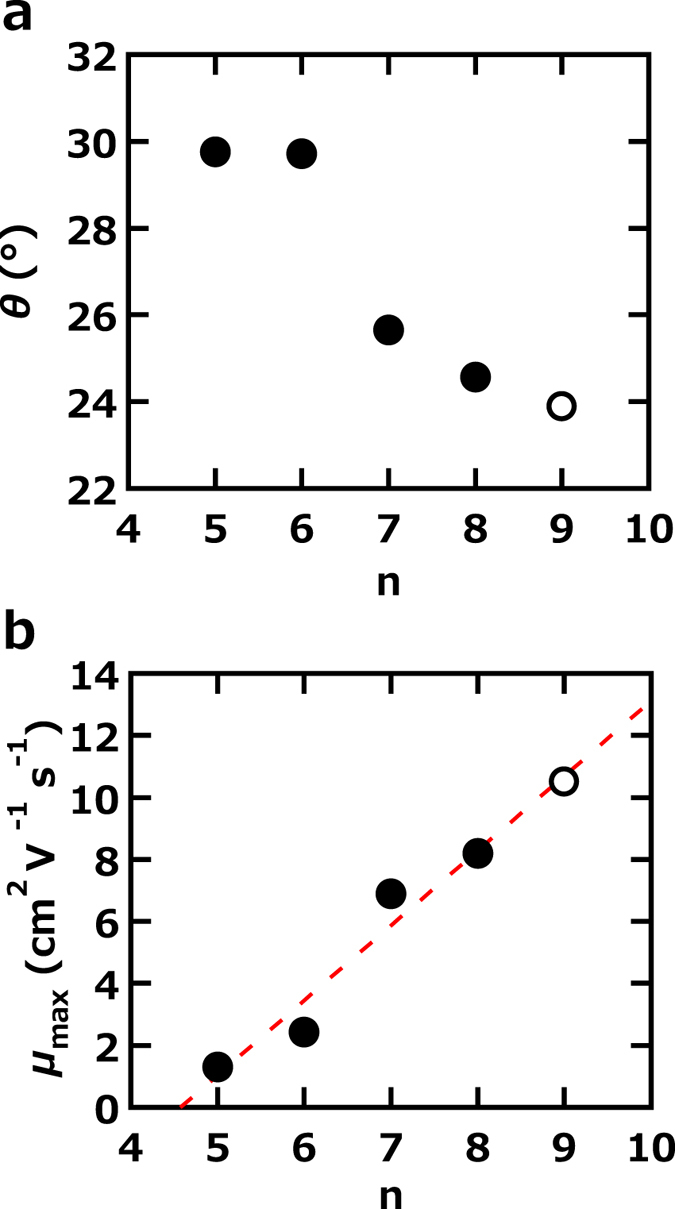
(**a**)*θ*- n and (**b**) *μ* – n plots for [n]phenacene single-crystal FETs. Open circles refer to [9]phenacene, while solid circles refer to other phenacenes (n = 5 – 8).

The characterization of [9]phenacene was fully performed using its thin films and single crystals. The MALDI-TOF-MS spectrum and the <*d*_001_ > (= < 1/|c^*^| > ) of [9]phenacene provide striking evidence for the successful synthesis, because NMR is not a useful tool for a characterization due to the compound’s low solubility. In particular, we provided the value of <*d*_001_ > as an effective indicator for the identification of the number of benzene rings (or n) in a phenacene molecule. The linear relationship between the <*d*_001_ > and n (Fig. 2c) clearly holds because of the nearly identical inclining-angle among phenacene molecules. The orientation of the molecule in the crystals is the same as that of other phenacenes. The van der Waals interaction between phenacene molecules is probably produced by both the π-π interaction and CH…π interactions, which would be expected to result in the herringbone stacking of phenacene molecules in their crystals. With increasing n, the contribution of the π-π interaction should increase because of the extended benzene network (or the increase in the number of C atoms), while the CH…π interaction should slowly decrease because of the decrease in the ratio of H atoms to C atoms (0.636 for picene (n = 5) to 0.579 for [9]phenacene (n = 9)). The larger π-π interaction and smaller CH…π interaction probably lead to a 2D-layered packing structure with aligned molecular planes from herringbone structure. Actually, as seen from Fig. 7a, the *θ* decreases slowly, indicating a slow change toward forming π-π stacking structure. However, we assumed that [9]phenacene crystals still maintained the herringbone structure because of the similar lattice constants of *a* and *b* to those of picene (see Table 1).

As described in Results, the energy diagram of [9]phenacene shows a deeper valence band edge and narrower band gap than other phenacenes (n = 5 – 8). The narrower band gap in [9]phenacene can be approximately explained by its benzene network, which is more extended than that in other phenacenes (n = 5 – 8), but the W-shape structure in the phenacene molecule does not lead to a rapid extension of the π-framework with increasing n, which leads to a slow decrease in the band gap. This results in higher stability in phenacene molecules than in acenes, which exhibit a rapid decrease in band gap^31^. The chemical stability may decrease the number of trap states originating in the decomposition of the molecule. This would lead to its high channel transport and high FET performance.

Figure 7b shows the *μ* – n plot for the phenacene single-crystal FET, in which the *μ* corresponds to the highest *μ* of the phenacene single-crystal FETs with SiO_2_ gate dielectrics. The *μ* increases straightforwardly with increasing n. This implies that the extended π-network is effective for channel transport because the increase in n enhances the overlap (π - π interaction) between 2p_z_ orbitals in carbon atoms. Furthermore, the <*μ* > value, 5.5(8) cm^2^ V^−1^ s^−1^, of [9]phenacene single-crystal FET with PZT gate dielectric was higher than that, 1.6(4) cm^2^ V^−1^ s^−1^, of [8]phenacene single-crystal FET with PZT^22^, indicating that the *μ* substantially increases with increasing number of benzene rings even if the gate dielectric was changed from SiO_2_ to PZT. However, it should be noted that the plot of *μ* – n is not prepared based on the averaged *μ* value (<*μ* > ). For strictly confirming the increase in mobility against n, the <*μ* > - n plot must be prepared by evaluating the <*μ* > value for picene, [6]phenacene and [7]phenacene single-crystal FETs since the <*μ* > values for these FETs have not yet been obtained. Nevertheless, the *μ*-n plot using the highest *μ* value would be statistically significant because many devices are investigated for searching for the highest *μ* value.

The decrease in *θ* versus n, shown in Fig. 7a, must cause the increase in π-π interaction. This is also the origin of the increase in *μ versus* n. Thus, the extension of the π-network in phenacene provides multiple advantages for increasing *μ*. From the graph shown in Fig. 7b, the even-odd effect of the number of benzene rings is negligible. As a consequence, we suggest that more extended phenacene molecules (n > 9) must be synthesized and used in FET devices to produce higher-performance FETs. For at least the phenacene molecules, the extension of the π-framework is still a key to the realization of higher *μ* values.

## Methods

### Experimental details of synthesis and characterization of [9]phenacene

The preparation process for the [9]phenacene molecule reported in Results is more fully described in this section, which includes the actual experimental procedures. A solution of KOH (1.7 g in 1.7 ml of water) was added to a mixture of aldehyde **1** (1.56 g, 1.0 mmol) and phosphonium salt **2** (4.02 g, 1.0 mmol) in 30 ml of CH_2_Cl_2_. The mixture was vigorously stirred at room temperature for 4 hours, and the organic layer was collected, washed with water, and dried with CaCl_2_. The solvent was evaporated under reduced pressure. The residue was chromatographed on silica gel using CH_2_Cl_2_ to afford compound **3** as a mixture of *E*- and *Z*-diastereomers. Compound **3** was dissolved in 1.9 l of cyclohexane, and 260 mg of iodine was added. The solution was photo-irradiated with a 450-W high-pressure mercury arc lamp using a hand-crafted flow reaction apparatus^34^. During photo-irradiation, the solution flowed at a rate of 5 ml min^−1^. The solvent was evaporated under reduced pressure, and the residue was washed with methanol to afford 1-methylchrysene **4** (1.85g, 80% for the two steps). The ^1^H NMR spectrum of the obtained sample (shown in Figure S7 of Supplementary Information) was identical to that previously reported^21^. The reaction process is shown in Figure S6a of the supplementary information.

A mixture of 1-methylchrysene **4** (1.85 g, 7.64 mmol), NBS (1.36 g, 7.64 mmol), and BPO (70%) (132 mg, 0.38 mmol) in CCl_4_ (130 ml) was refluxed for 17 hours. After cooling to room temperature, the precipitate was collected, and washed with toluene and then MeOH to afford 1-(bromomethyl)chrysene **5** (1.83 g, 74%). The ^1^H NMR spectrum (Figure S8 in Supplementary Information) was identical to that previously reported^21^. The reaction process is shown in Figure S6b of Supplementary Information.

A mixture of 1-(bromomethyl)chrysene **5** (867 mg, 7.70 mmol) and hexamethylenetetramine (454 mg, 3.20 mmol) in 100 ml of CHCl_3_ was refluxed for 16.5 hours. The solvent was evaporated under reduced pressure. The residue was suspended in a mixture of acetic acid (50 ml) and water (20 ml), and it was refluxed for 20 hours. To the resulting mixture was added 50 ml of water, and the solid was collected and washed with water. The solid was triturated with a boiling toluene-CHCl_3_ mixture for ~ 0.5 hour. The insoluble material was filtered off, and the filtrate was concentrated under reduced pressure to afford 1-chrysenecarbaldehyde **6** (486 mg, 70%). Sample **6** was purified with preparative thin-layer chromatography (silica gel, toluene), followed by recrystallization from toluene. Physical data for **6**: Pale brown crystals, mp 229–230 °C. ^1^H NMR (600 MHz, CDCl_3_) δ_H_: 10.52 (s, 1H), 9.38 (d, 1H, *J* = 9.5 Hz), 9.04 (d, 1H, *J* = 8.4 Hz), 8.88 (d, 1H, *J* = 9.5 Hz), 8.79 (d, 1H, *J* = 8.2 Hz), 8.66 (d, 1H, *J* = 9.0 Hz), 8.09 (dd, 1H, *J* = 7.1, 1.1 Hz), 8.02 (d, 1H, *J* = 9.0 Hz), 8.00 (d, 1H, *J* = 7.9 Hz), 7.82 (dd, 1H, *J* = 8.2, 7.1 Hz), 7.74 (ddd, 1H, *J* = 8.2, 7.1, 1.1 Hz), 7.67 (ddd, 1H, *J* = 7.9, 6.8, 1.0 Hz). ^13^C NMR (150 MHz, CDCl_3_) δ_C_: 193.7, 135.6, 132.3, 131.7, 131.1, 130.3, 130.2, 129.8, 128.7, 128.5, 128.2, 128.0, 127.2, 127.0, 125.8, 124.6, 123.4, 123.1, 121.1. IR (neat) ν_max_ 2710, 1684, 799, 772, 748 cm^−1^. Elemental Analysis: 89.01 for C and 4.56 for H (calculated values: 89.04 for C and 4.72 for H). ^1^H and ^13^C NMR spectra of compound **6** are shown in Figure S9 of Supplementary Information. This reaction process is shown in Figure S6c of Supplementary Information.

A solution of 1-(bromomethyl)chrysene **5** (963 mg, 3.0 mmol) and triphenylphosphine (868 mg, 3.3 mmol) in *N*,*N*-dimethylformamide (DMF) (50 ml) was stirred at room temperature for 17 hours. The solvent was removed under reduced pressure, and the residue was washed with toluene to afford phosphonium salt **7** (1.51 g, 86%). The ^1^H NMR spectrum (Figure S10 of Supplementary Information) was identical to that previously reported^21^. The reaction process is shown in Figure S6d of Supplementary Information.

A solution of tetrabutylammonium hydroxide (1 ml, 1 mol l^−1^ in methanol) was added to a solution of 1-chrysenecarbaldehyde **6** (100 mg, 0.39 mmol) and (1-chrysenyl)methyltriphenylphosphonium bromide **7** (228 mg, 0.50 mmol) in 50 ml of CH_2_Cl_2_, and the mixture was stirred at room temperature for 1 hour. The precipitate formed was collected and washed successively with CHCl_3_ and MeOH to afford 1,2-bis-(1-chrysenyl)ethene **8** (169 mg, 90%). This reaction is shown in Figure S6e. The obtained product was used in the next step without purification.

1,2-Bis-(1-chrysenyl)ethene **8** (60 mg, 0.12 mmol) was dissolved in 250 ml of bromobenzene by heating at 130–140 °C, and a small portion of iodine was added. The solution was photo-irradiated with six 15-W black-light lamps at 130–140 °C. The precipitate was collected and washed successively with bromobenzene and MeOH to afford [9]phenacene as off-white fine plates (51 mg, 89%). The sample was purified by sublimation under flowing argon, which produced transparent single crystals. Physical data for [9]phenacene: mp 490 °C (determined by differential scanning calorimetry). IR (neat) ν_max_: 3087, 3049, 3014, 1442, 1430, 1286, 1025, 944, 866, 804, 774, 740 cm^−1^. *m*/*z* of MALDI-TOF MS: 477.97 (100%), 478.98 (49.4%) 480.03 (10.6%), 481.05 (1.4%) (see Fig. 2a). Elemental Analysis: 95.36 for C and 4.35 for H (calculated values: 95.37 for C and 4.63 for H). The reaction process is shown in Figure S6f of Supplementary Information.

The XRD patterns of the crystalline powder and a single crystal of [9]phenacene were measured using a RIGAKU SMARTLAB-PRO with Cu Kα source (wavelength of 1.5418 Å) and the AFM images of a thin film and single crystal were recorded with a measurement system (SII Nano Technology SPA400). The preparation of single crystals is described in the subsequent section.

### Preparation of thin films and single crystals of [9]phenacene

Thin films of [9]phenacene were formed by thermal deposition of [9]phenacene under a vacuum of 10^−6^ Torr. The temperature of the substrate was maintained at room temperature. The thin films used for the measurement of XRD and AFM were formed on a Si/SiO_2_ substrate with the SiO_2_ surface treated with HMDS. Single crystals of [9]phenacene were made using the physical vapour transport (PVT) method. The equipment was shown in Fig. 5 of ref. 23. The method for obtaining [9]phenacene single crystals is substantially the same as the description in ref. 30. The temperature of the hot zone where the source sample was placed was 763 K, while the temperature for cold zone where the single crystals were obtained was 573 K. The Ar carrier gas flowed at a rate of 50 ml min^−1^ in the PVT method. The obtained single crystals were plate-shape and transparent (see Figure S3b and d).

### Fabrication of FET devices using thin films and single crystals

The surfaces of the SiO_2_ gate dielectrics used for [9]phenacene thin-film FETs were treated with HMDS to produce a hydrophobic surface. The details of method are described elsewhere^20^. However, the surfaces of the high-*k* gate dielectrics used for all [9]phenacene FETs and SiO_2_ used for [9]phenacene single-crystal FETs were coated with 50 nm thick parylene. This surface is also hydrophobic in comparison with a bare SiO_2_ surface. The method of parylene-coating is described elsewhere^35^.

Thin films of [9]phenacene were formed on gate dielectrics by thermal deposition under a vacuum of 10^−6^ Torr, with the substrate kept at room temperature during thermal deposition. However, the single crystals were simply placed on the gate dielectrics. Au source/drain electrodes 50 and 86 nm thick were formed on the thin films and single crystals by thermal evaporation under 10^−6^ Torr. F_4_TCNQ 3 nm thick was inserted between thin films or single crystals and source/drain electrodes to reduce contact resistance. All FET devices used a top-contact structure. The device structures of the [9]phenacene FETs with solid gate dielectrics are shown in Figs 4a,5a and 6a. EDL polymer sheet was fabricated using [1-butyl-3-methylimidazolium][hexafluorophosphate] (bmim[PF_6_]) and poly(vinylidene fluoride-co-hexafluoropropyrene). Details of its synthesis are given in ref. 21. As described in the Results section, the gate voltage was applied from a side gate electrode in [9]phenacene thin-film EDL FETs, *i.e.*, this device is a side-gate top-contact structure (see Figure S4a), while the gate voltage was applied through the Au plate in the [9]phenacene single-crystal EDL FETs, *i.e.*, this device is a top-gate top-contact structure (see Figure S5a)

The FET characteristics were measured using a semiconductor parameter analyser (Agilent B1500A) for the FET devices in Ar-filled glove box. The capacitance was measured using a precision LCR meter (Agilent E4980A). The *C*_o_ used for analysis of FET performance was determined by extrapolation of the capacitance measured at 20 Hz – 1 kHz to 0 Hz. The *C*_o_ of each gate-dielectric at 0 Hz is as follows: *C*_o_ = 9.46 nF cm^−2^ for parylene-coated SiO_2_, C_o_ = 8.34 nF cm^−2^ for HMDS-coated SiO_2_, *C*_o_ = 27.6 nF cm^−2^ for parylene-coated ZrO_2_, *C*_o_ = 40.6 nF cm^−2^ for parylene-coated PZT, and *C*_o_ = 8.01 *μ*F cm^−2^ for EDL polymer sheet. The methods of gating and measurement of *I*_D_ are shown schematically in Fig. 4a, S4a, 5a, 6a and S5a.

## Additional Information

**How to cite this article**: Shimo, Y. *et al.* Synthesis and transistor application of the extremely extended phenacene molecule, [9]phenacene. *Sci. Rep.* **6**, 21008; doi: 10.1038/srep21008 (2016).

## Supplementary Material

Supplementary Information

## Figures and Tables

**Table 1:** Lattice constants of [n]phenacene molecules (n = 5 – 9); picene refers to [5]phenacene.

	*a* [Å]	*b* [Å]	*c* [Å]	*β*[°]	Ref.
Picene	8.472(2)	6.170(2)	13.538(7)	90.81(4)	29
[6]phenacene	12.130(1)	7.9416(7)	15.401(1)	93.161(8)	30
[7]phenacene	8.4381(8)	6.1766(6)	17.829(2)	93.19(1)	30
[8]phenacene	8.842(2)	6.043(1)	19.896(4)	92.92(3)	22
[9]phenacene	8.844(5)	6.127(3)	22.47(1)	92.72(5)	–

**Table 2:** FET parameters of [9]phenacene single-crystal FET with SiO_2_ gate dielectric.

sample	*μ* (cm^2^V^−1^s^−1^)	|*V*_th_| (V)	ON/OFF	*S* (V/decade)	*L* (μm)	*W* (μm)
#1	5.5	22.2	1.3 × 10^8^	0.84	50	455
#2	7.0	29.4	8.3 × 10^7^	0.81	100	502
#3	8.9	26.0	8.7 × 10^7^	0.81	150	518
#4	7.5	32.2	5.4 × 10^7^	0.89	200	543
#5	8.2	37.0	9.8 × 10^6^	1.3	200	225
#6	8.8	29.9	2.7 × 10^7^	0.82	285	341
#7	7.9	27.9	2.7 × 10^7^	1.1	450	621
#8	8.7	22.0	1.0 × 10^9^	0.71	100	400
#9	9.1	22.0	7.4 × 10^8^	0.79	135	392
#10	10.5	16.5	5.3 × 10^8^	0.90	200	378
average	8(1)	27(6)	3(4) × 10^8^	0.9(2)	–	–

The parameters were determined from the forward transfer curves.

**Table 3:** FET parameters of [9]phenacene single-crystal FET with ZrO_2_ gate dielectric.

sample	*μ*(cm^2^V^−1^s^−1^)	|*V*_th_| (V)	ON/OFF	*S* (V/decade)	*L* (μm)	*W* (μm)
#1	11.9	2.37	1.4 × 10^6^	0.261	200	366
#2	17.9	2.12	2.9 × 10^7^	0.212	150	449
#3	4.96	2.16	9.1 × 10^5^	0.309	50	250
#4	6.82	0.87	2.3 × 10^6^	0.172	100	647
#5	10.1	1.29	9.3 × 10^6^	0.153	285	1185
average	10(5)	1.8(6)	1(1) × 10^7^	0.22(6)	–	–

The parameters were determined from the forward transfer curves.
