# Supplementary material for: Synthesis and transistor application of the extremely extended phenacene molecule, [9]phenacene
Source: Sci Rep. 2016 Feb 19;6:21008. doi: 10.1038/srep21008 (PMC4759550; doi:10.1038/srep21008)
Supplement: Supplementary Information [file srep21008-s1.pdf]

Supplementary Information

Synthesis and transistor application of the extremely extended phenacene molecule, [9]phenacene

Yuma Shimo[1], Takahiro Mikami[1], Shino Hamao[2], Hidenori Goto[2], Hideki Okamoto[3], Ritsuko Eguchi[2], Shin Gohda[4], Yasuhiko Hayashi[1], Yoshihiro Kubozono[2, 5,*]

[1]Department of Electric and Electronic Engineering, Okayama University, Okayama 700-8530, Japan

[2]Research Laboratory for Surface Science, Okayama University, Okayama 700-8530, Japan

[3]Department of Chemistry, Okayama University, Okayama 700-8530, Japan

[4]NARD Co. Ltd. Amagasaki 660-0805, Japan

[5]Research Centre of New Functional Materials for Energy Production, Storage and Transport, Okayama University, Okayama 700-8530, Japan

Table of Contents

① TOF mass spectral data of [9]phenacene. Table S1 lists the observed and calculated values of *m* / *z*.

② FET parameters of [9]phenacene thin-film FETs with SiO_2 gate dielectrics (Table S2). Amorphous thin films were used as active layers.

③ FET parameters of [9]phenacene thin-film FETs with SiO_2 gate dielectrics (Table S3). Polycrystalline thin films were used as active layers.

④ FET Parameters of [9]phenacene thin-film EDL FETs (Table S4).

⑤ FET Parameters of [9]phenacene single-crystal FETs with SiO_2 gate dielectric (Table S5). The FET parameters are evaluated from the reverse transfer curves.

⑥ FET parameters of [9]phenacene single-crystal FETs with PZT gate dielectrics (Table S6).

⑦ FET Parameters of [9]phenacene single-crystal EDL FETs (Table S7).

⑧ XRD pattern of polycrystalline [9]phenacene powder sample (Figure S1)

⑨ *c* – n plot of [n]phenacenes determined from XRD patterns of polycrystalline powder samples (Figure S2).

⑩ Optical and AFM images of [9]phenacene thin film and a single crystal (Figure S3).

⑪ FET characteristics of [9]phenacene thin-film EDL FET (Figure S4).

⑫ FET characteristics of [9]phenacene single-crystal EDL FET (Figure S5).

⑬ Synthetic route to [9]phenacene molecule (Figure S6).

⑭ ^{1}H NMR of 1-methylchrysene 4 (Figure S7).

⑮ ^{1}H NMR of 1-(bromomethyl)chrysene 5 (Figure S8)

⑯ ^{1}H NMR and 13C NMR of 1-chrysenealdehyde 6 (Figure S9)

⑰ ^{1}H NMR of (1-chrysenyl)methyltriphenylphosphonium bromide 7 (Figure S10)

Table S1. TOF mass spectral data of [9]phenacene ($C_{38}H_{22}$) sample. The 'calculated' and 'observed' correspond to the values calculated considering natural abundance of C and H, and the experimental values obtained from the TOF mass spectrum shown in Figure 2a.

**Observed**		**Calculated**	
m/*z*	Relative Intensity	*m*/*z*	Relative Intensity
477.97	100%	478.17	100%
478.98	49.4%	479.18	41.4%
480.03	10.6%	480.18	8.3%
481.05	1.4%	481.18	1.1%

Table S2. FET parameters of [9]phenacene thin-film FETs with SiO_2 gate dielectrics. The parameters were determined from the forward transfer curves. Amorphous thin films were used for active layers.

**Sample**	**μ ($cm^2V^{-1}s^{-1}$)**	**$\lvert V_{th} \rvert$ (V)**	**ON/OFF**	**S (V decade^{-1})**	**L (μm)**	**W (μm)**
**#1**	8.15×10^{-2}	49.5	2.33×10^{4}	8.31	200	500
**#2**	9.57×10^{-2}	42.4	5.15×10^{3}	20.3	350	500
**#3**	9.53×10^{-2}	50.2	9.53×10^{3}	20.7	350	500
**#4**	1.50×10^{-1}	49.1	1.24×10^{4}	9.68	450	500
**#5**	1.20×10^{-1}	46.2	1.46×10^{4}	9.69	450	500
**#6**	1.48×10^{-1}	47.2	1.39×10^{4}	10.1	450	500
**average**	$1.2(3)\times10^{-1}$	47(3)	$1.3(6)\times10^{4}$	13(5)		

Table S3. FET parameters of [9]phenacene thin-film FETs with SiO_2 gate dielectrics. The parameters were determined from the forward transfer curves. Polycrystalline thin films were used for active layers.

sample	μ ($cm^2V^{-1}s^{-1}$)	$\|V_{th}\|$ (V)	ON/OFF	S (V/decade)	L (μm)	W (μm)
#1	1.18	53.0	2.70×10^7	3.2	200	500
#2	0.975	40.1	2.10×10^7	5.8	350	500
#3	0.685	44.6	1.25×10^7	5.0	350	500
#4	1.69	42.4	2.61×10^7	3.9	450	500
#5	1.25	40.5	2.05×10^7	1.0	450	500
#6	1.24	34.1	2.50×10^7	1.0	450	500
**average**	1.2(3)	42(6)	$2.2(5)\times10^7$	3(2)		

Table S4. FET parameters of [9]phenacene thin-film EDL FETs. The parameters refer to the averaged values evaluated from the forward and reverse transfer curves.

Sample	μ ($cm^2V^{-1}s^{-1}$)	$\lvert V_{th} \rvert$ (V)	ON/OFF	S (V $decade^{-1}$)	L (μm)	W (μm)
**#1**	0.93	1.90	8.7×10^2	0.73	100	410
**#2**	0.63	1.66	4.3×10^2	0.83	100	307
**#3**	1.15	2.06	1.8×10^3	0.61	100	364
**#4**	1.07	1.89	2.1×10^2	0.63	100	545
**average**	0.9(2)	1.9(2)	8(7) $\times 10^2$	0.7(1)		

Table S5. FET parameters of [9]phenacene single-crystal FET with SiO_2 gate dielectrics. The parameters are evaluated from the reverse transfer curves.

**Sample**	***μ*** **($cm^2V^{-1}s^{-1}$)**	**|V_{th}|** **(V)**	**ON/OFF**	***S*** **(V $decade^{-1}$)**	***L*** **(μm)**	***W*** **(μm)**
**#1**	9.60	42.5	1.4×10^8	1.23	50	455
**#2**	10.54	43.0	8.1×10^7	1.01	100	502
**#3**	11.63	35.3	7.9×10^7	1.16	150	518
**#4**	11.17	44.7	4.7×10^7	1.44	200	543
**#5**	11.70	47.7	1.2×10^7	1.76	200	225
**#6**	11.48	38.8	2.6×10^7	1.34	285	341
**#7**	11.81	41.4	2.1×10^7	1.77	450	621
**#8**	9.37	24.8	1.9×10^9	0.83	100	400
**#9**	10.57	27.1	5.0×10^8	0.88	135	392
**#10**	11.61	20.7	7.4×10^8	0.87	200	378
**average**	10.9(9)	37(9)	$4(6)\ \times10^8$	1.2(3)	-	-

Table S6. FET parameters of [9]phenacene single-crystal FET with PZT gate dielectrics. The parameters were determined from the forward transfer curves.

Sample	μ ($cm^2V^{-1}s^{-1}$)	$\|V_{th}\|$ (V)	ON/OFF	S (V $decade^{-1}$)	L (μm)	W (μm)
**#1**	4.59	0.992	1.1×10^{3}	1.7	50	302
**#2**	6.22	1.40	1.7×10^{7}	0.19	100	303
**#3**	5.56	1.51	3.8×10^{6}	0.23	150	287
**average**	5.5(8)	1.3(3)	7(9) $\times10^{6}$	0.7(8)	-	-

Table S7. FET parameters of [9]phenacene single-crystal EDL FETs. The parameters refer to the averaged values evaluated from the forward and reverse transfer curves.

**Sample**	μ ($cm^2V^{-1}s^{-1}$)	$\lvert V_{th} \rvert$ (V)	**ON/OFF**	S (V $decade^{-1}$)	L (μm)	W (μm)
**#1**	0.341	1.71	2.5×10^6	0.133	135	392
**#2**	1.51	1.06	4.1×10^5	0.131	100	400
**#3**	1.66	1.91	4.3×10^5	0.179	50	302
**average**	1.2(7)	1.6(4)	$1(1)\ \times10^6$	0.15(3)	-	-

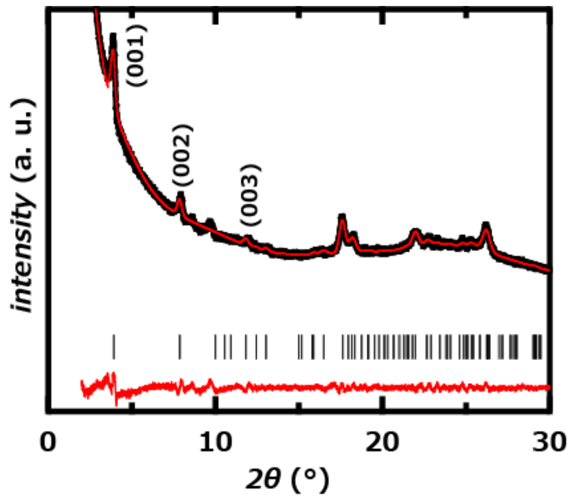

Figure S1. XRD pattern of polycrystalline [9]phenacene powder sample.

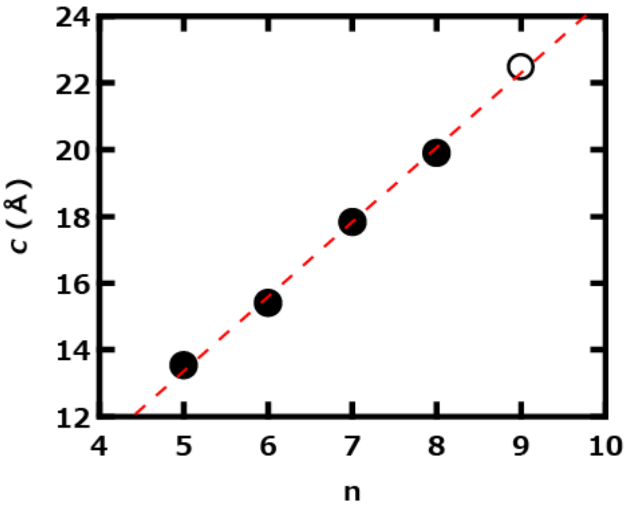

Figure S2. *c* – n plot of phenacenes. Dimension *c* was evaluated from the XRD of polycrystalline powder samples (see Table 1).

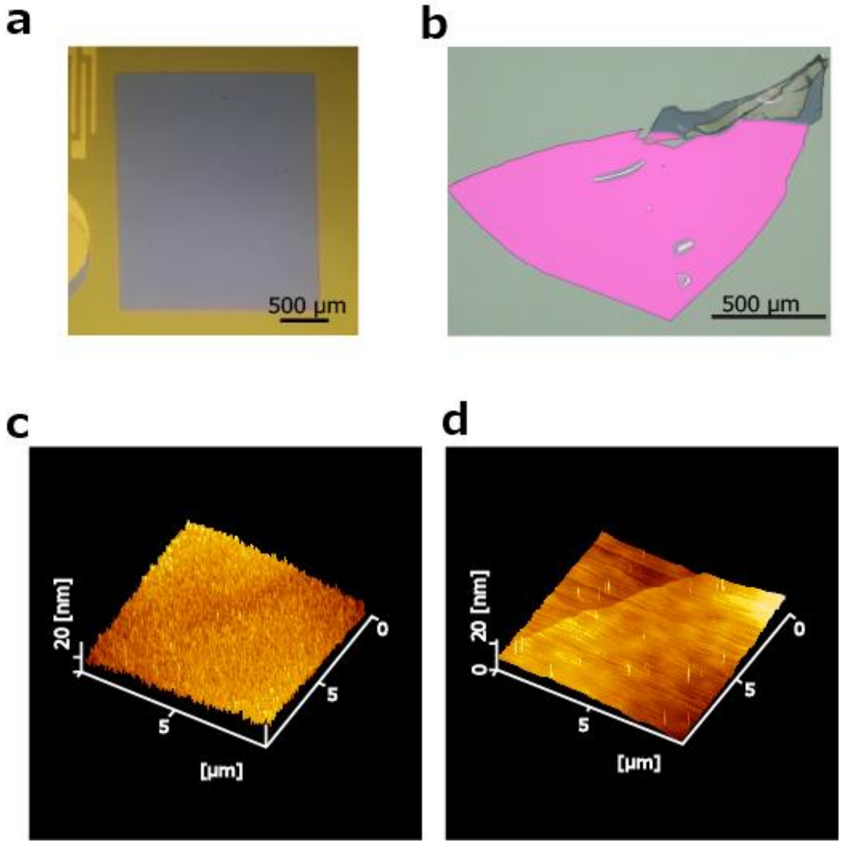

Figure S3. Optical microscope images of (a) thin film and (b) single crystal of [9]phenacene. AFM images of (c) thin film and (d) a single crystal of [9]phenacene.

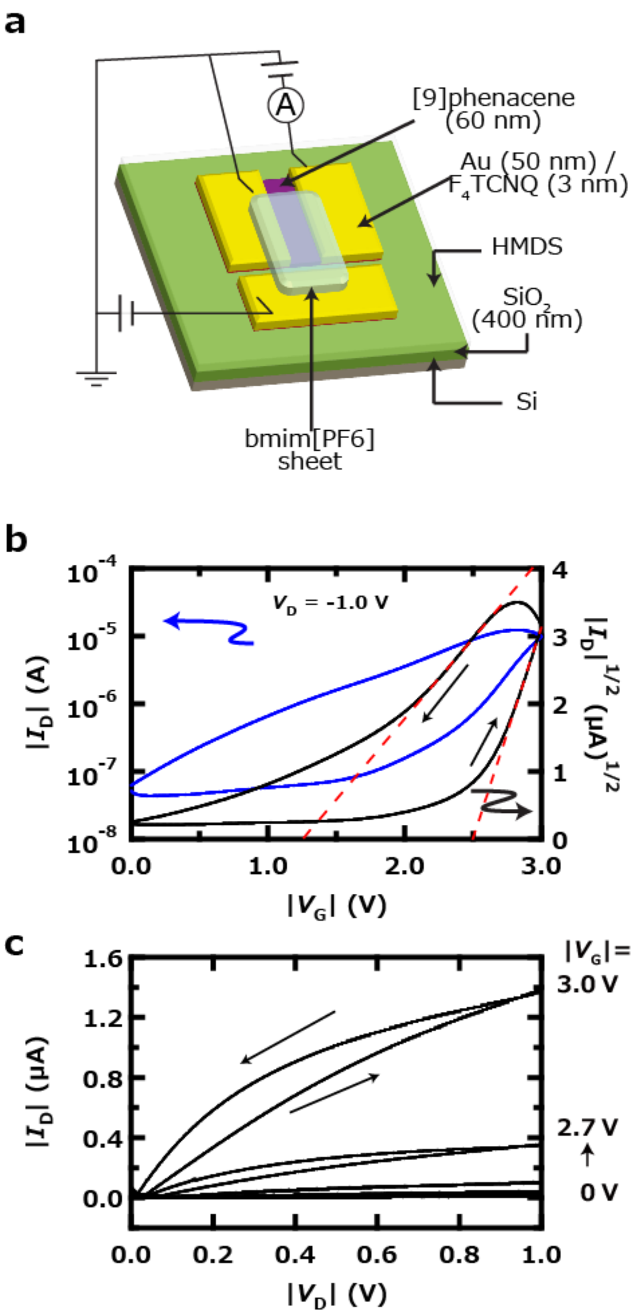

Figure S4. (a) Schematic representation of [9]phenacene thin-film FET with an EDL capacitor. (b) Transfer and (c) output curves of [9]phenacene thin-film FETs with an EDL capacitor.

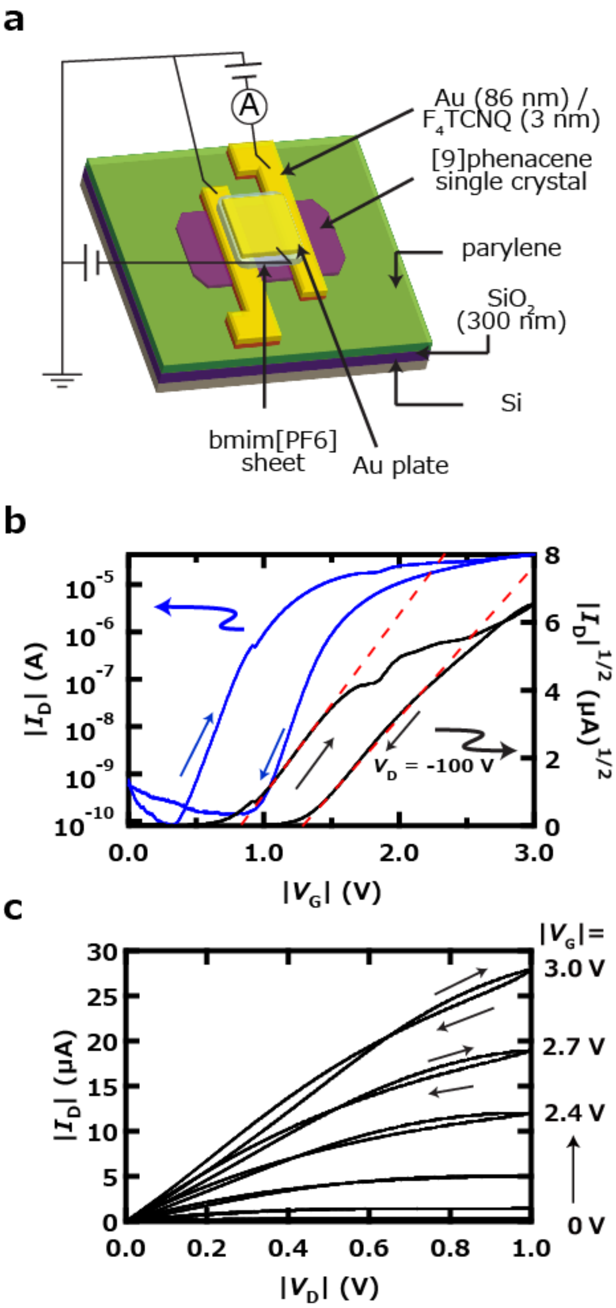

Figure S5. (a) Schematic representation of [9]phenacene single-crystal FET with an EDL capacitor. (b) Transfer and (c) output curves of a [9]phenacene single-crystal FET with an EDL capacitor.

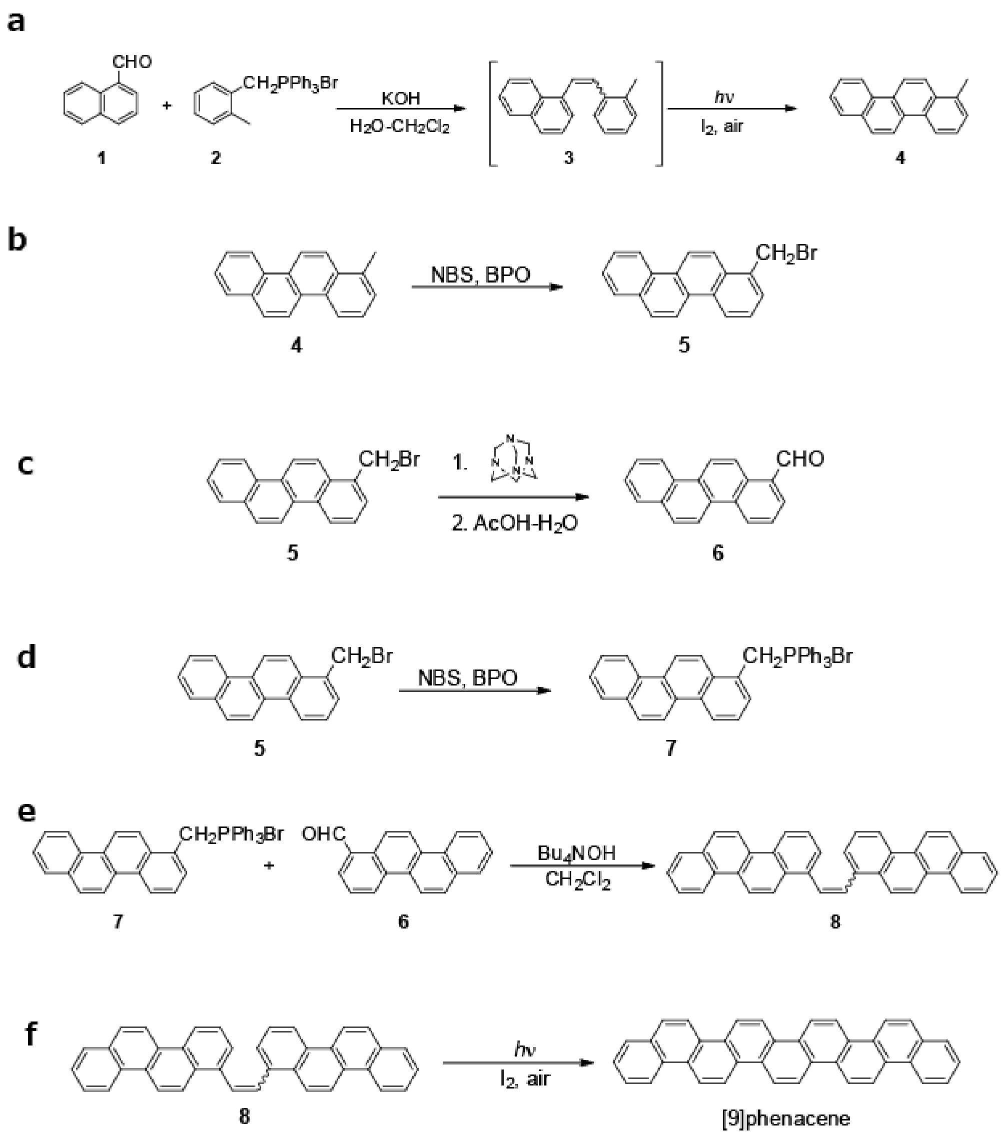

Figure S6. Details of synthetic protocol for each step.

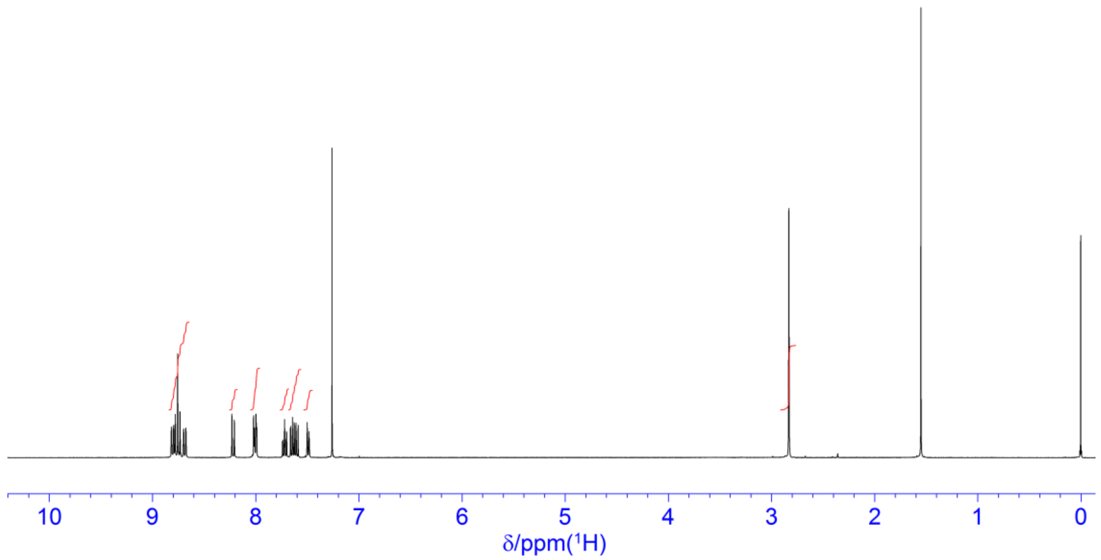

Figure S7. ¹H NMR spectrum of 1-methylchrysene **4** (400 MHz, $CDCl_3$).

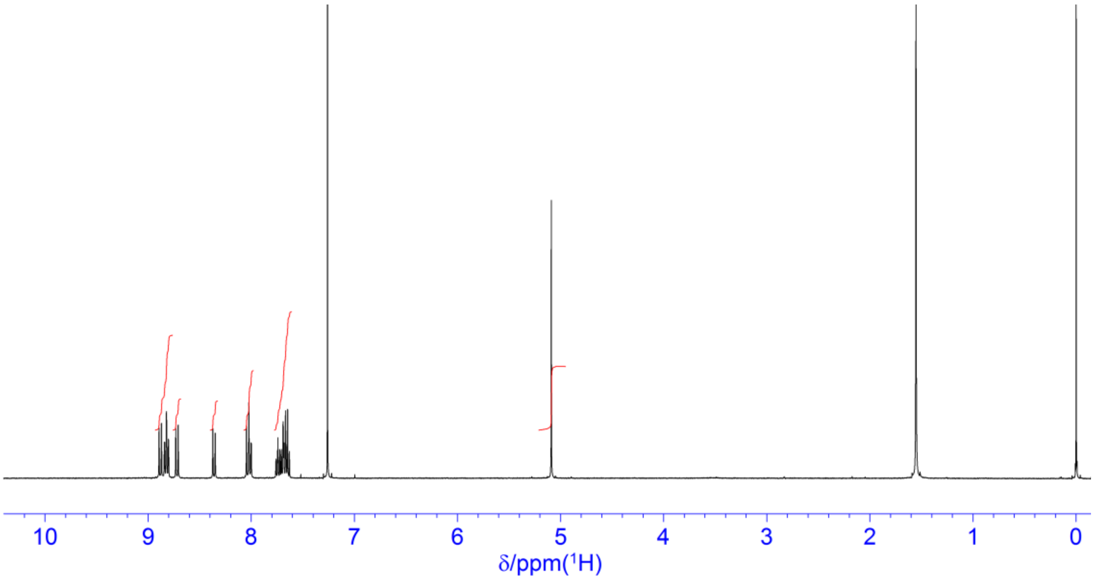

Figure S8. ^{1}H NMR spectrum of 1-(bromomethyl)chrysene **5** (400 MHz, $CDCl_3$).

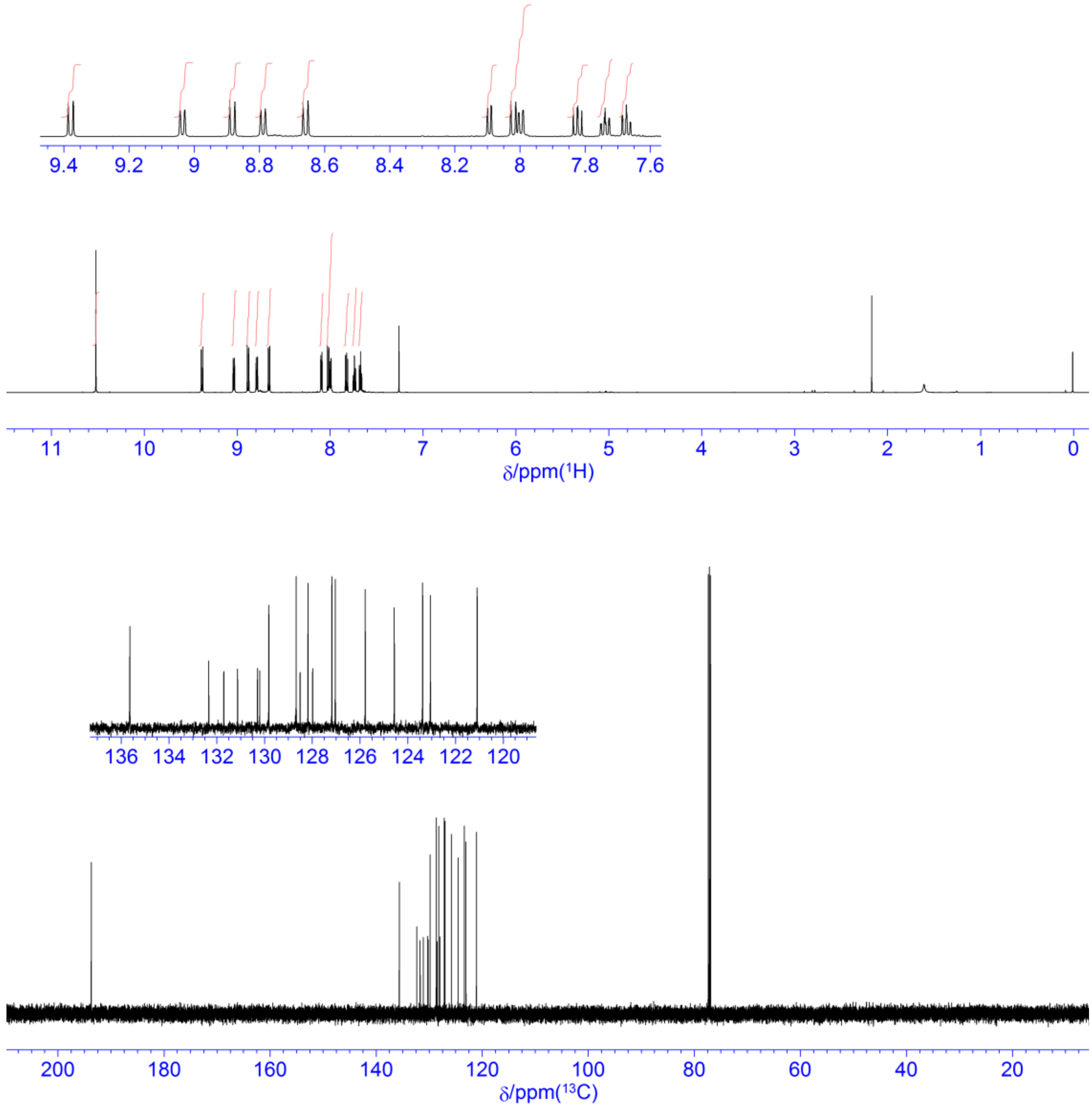

Figure S9. ^{1}H (upper, 600 MHz) and ^{13}C (lower, 150 MHz) NMR spectra of 1-chrysenecarbaldehyde **6** ($CDCl_3$).

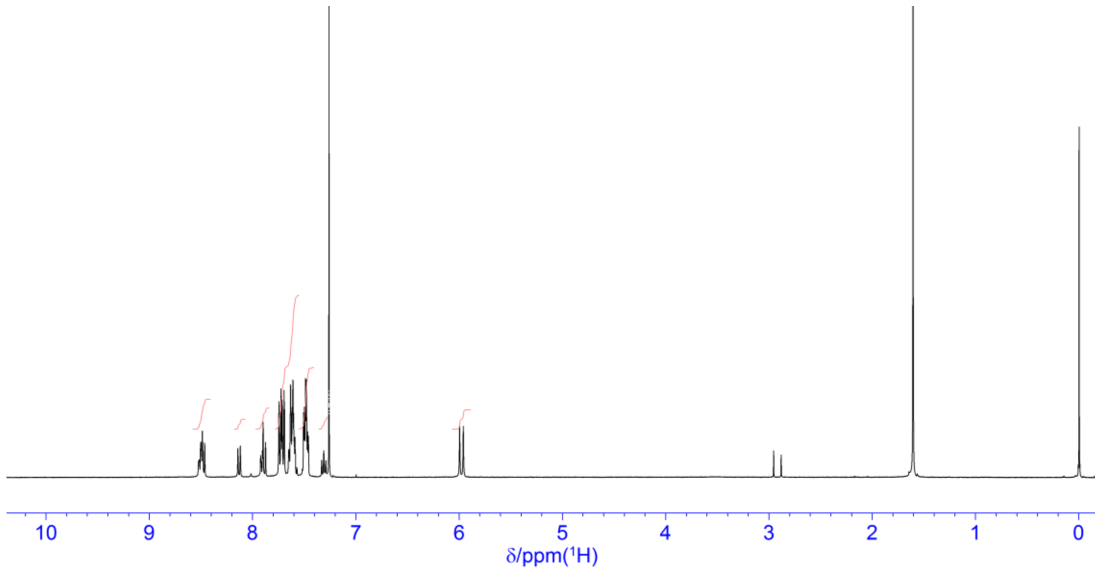

Figure S10. ^{1}H NMR spectrum of (1-chrysenyl)methyltriphenylphosphonium bromide **7** (400 MHz, $CDCl_3$).
